# Supplementary material for: Quality of basic emergency obstetric and newborn care services from patients’ perspective in selected public health centers in Addis Ababa, Ethiopia 2022: A cross-sectional study
Source: PLoS One. 2025 Apr 3;20(4):e0320729. doi: 10.1371/journal.pone.0320729 (PMC11967978; doi:10.1371/journal.pone.0320729)
Supplement: S1 File — (DOCX) [file pone.0320729.s001.docx]

**English Version Questionnaire**

1. **Socio-demographic factors**

| NO | Questions and filter | Codding category | Skip to |
| --- | --- | --- | --- |
|  | How old are you? | ……………………………yrs. |  |
|  | Residence | 1. Urban 2. Rural |  |
|  | What is your educational level? | 1. No formal education 2. Primary level 3. Secondary level 4. Diploma 5. Degree and above |  |
|  | What is your occupation? | 1. Governmental 2. Private 3. Daily Laborer 4. House wife 5. Other (specify….) |  |
|  | What is your marital status? | 1. Single 2. Married 3. Divorced 4. Widowed | If 1, 3, or 4 skip  to 1.08 |
|  | What is your husband’s education level? | 1. No formal education 2. Primary level 3. Secondary level 4. Diploma 5. Degree and above |  |
|  | What is your husband’s occupation? | 1. Governmental 2. Private (Merchant) 3. Daily laborer 4. Unemployed 5. Driver 6. Other (specify………….. |  |
|  | Average monthly household income in Ethiopian Birr. | ………………………. ETB |  |

| **Obstetric history** | | | |
| --- | --- | --- | --- |
|  | Gravidity | …………………… |  |
|  | ANC follow up | 1. Yes 2. No |  |
|  | Wanted status of current Pregnancy | 1. Wanted 2. Unwanted |  |
|  | Type of visit | 1. Planned(direct) 2. Referred |  |
|  | Mode of transportation | 1. Ambulance 2. Public transportation 3. Other specify………. |  |
|  | How long did you wait in this hospital before receiving care from health providers for this last delivery? | 1. <15 minute 2. 15-30 minute 3. 30-1 hour 4. > 1 hour |  |
|  | Presence of companion during stay | 1. Yes 2. No |  |
|  | Mode of Delivery | 1. SVD 2. Assisted vaginal delivery 3. Abortion (spontaneous) |  |
|  | How was your health condition after giving birth/Abortion? | 1. Normal 2. With complication |  |
|  | Birth outcome after delivery | 1. Alive 2. Neonatal death 3. Still birth 4. Abortion | If 2, 3 or 4 skip  to 1.20 |
|  | Was there any health problem on your newborn baby? | 1. Yes 2. No |  |
|  | Have you had to pay for any services or products during your stay? | 1. Yes 2. No |  |

1. **Questions on quality**

| No | Question & Filter | Strongly  disagree | Disagree | Neutral | Agree | Strongly  Agree |
| --- | --- | --- | --- | --- | --- | --- |
|  |  |  |  |  |  |  |
|  | Received proper respect and courtesy by the health providers during examination. |  |  |  |  |  |
|  | The environment where you were laboring was comfortable. |  |  |  |  |  |
|  | Health workers examined thoroughly and made active follow up on the progress of labor. |  |  |  |  |  |
|  | Health providers asked permission before applying any procedures and  Examination |  |  |  |  |  |
|  | Health worker explained the labor progress to you by using your local and clear language. |  |  |  |  |  |
|  | Have you felt safe because different member of staff have given you similar advice or information about your condition. |  |  |  |  |  |
|  | Health workers spent enough time for examination. |  |  |  |  |  |
|  | Health workers verbally encouraged praised and reassured during the time of labor. |  |  |  |  |  |
|  | You got enough care and support during the time of labor. |  |  |  |  |  |
|  | It felt that the delivery/Abortion room has every material needed to provide good maternal and newborn care. |  |  |  |  |  |
|  | It appeared that the health providers look like competent and was confident on their work. |  |  |  |  |  |
|  | Felt that your privacy was kept in delivery/Abortion room. |  |  |  |  |  |
|  | You get enough care and support during the time of delivery/Abortion care. |  |  |  |  |  |
|  | Felt that there was enough number of health providers in the ward. |  |  |  |  |  |
|  | The health workers were available whenever you needed help. |  |  |  |  |  |
|  | Felt that there were sufficient rooms, beds and space for laboring and delivering mothers. |  |  |  |  |  |
|  | The wards kept an acceptable level of sanitation. |  |  |  |  |  |
|  | Did the labor and delivery ward have functional and clean toilet and shower room? |  |  |  |  |  |
|  | Received enough support from the staff in breast- feeding your baby immediately after birth. |  |  |  |  |  |
|  | Received counseling on how to take care of your baby |  |  |  |  |  |
|  | Your baby received enough care and support. |  |  |  |  |  |
|  | Receive adequate anti pain while MVA was performed? |  |  |  |  |  |
|  |  |  |  |  |  |  |

1. **Satisfaction**

| No | Questions | Level of satisfaction | | | | |
| --- | --- | --- | --- | --- | --- | --- |
|  |  | Strongly  dissatisfied | dissatisfied | neutral | satisfied | Strongly  Satisfied |
|  |  |  |  |  |  |  |
|  | The way staffs have treated you with respect and respected your personal wishes, culture, and religion. |  |  |  |  |  |
|  | Health professional respect for your privacy during your stay. |  |  |  |  |  |
|  | By the number of health worker in the labor and delivery, ward. |  |  |  |  |  |
|  | By health workers competency and their confidence on their job. |  |  |  |  |  |
|  | The communication between doctor, nurse and other health staff about your treatment and condition |  |  |  |  |  |
|  | The way staff involved you in decision about you and your baby condition. |  |  |  |  |  |
|  | By the overall Counseling that were given in your hospital stay |  |  |  |  |  |
|  | By the overall care and support, given during labor and delivery time. |  |  |  |  |  |
|  | By the care and support given for your newborn baby. |  |  |  |  |  |
